# Targeting the INCENP IN-box–Aurora B interaction to inhibit CPC activity *in vivo*

**DOI:** 10.1098/rsob.140163

**Published:** 2014-11-12

**Authors:** Florence H. Gohard, Daniel J. St-Cyr, Mike Tyers, William C. Earnshaw

**Affiliations:** 1 Wellcome Trust Centre for Cell Biology, Institute of Cell Biology, University of Edinburgh, Michael Swann Building, Kings Buildings, Mayfield Road, Edinburgh EH9 3JR, UK; 2 Institute for Research in Immunology and Cancer, Department of Medicine, Université de Montréal, Pavillon Marcelle-Coutu, 2950 chemin de Polytechnique, Montréal, Québec H3T 1J4, Canada

**Keywords:** chromosomal passenger complex, Aurora B, INCENP, mitosis, cytokinesis, cyclic peptide

## Abstract

The chromosome passenger complex (CPC) is an essential regulator of mitosis and cytokinesis. The CPC consists of Aurora B kinase, inner centromere protein (INCENP), and the targeting subunits survivin and borealin/Dasra B. INCENP is a scaffolding subunit for the CPC and activates Aurora B via its conserved IN-box domain. We show that overexpression of soluble IN-box in HeLa cells affects endogenous CPC localization and produces a significant increase in multinucleated and micronucleated cells consistent with CPC loss of function. The dominant-negative effect of soluble IN-box expression depends on residues corresponding to hINCENP W845 and/or F881, suggesting that these are essential for Aurora B binding *in vivo*. We then screened a targeted library of small (five to nine residues long) circular peptide (CP) IN-box fragments generated using split intein circular ligation of proteins and peptides (SICLOPPS) methodology. We identified a number of CPs that caused modest but reproducible increases in rates of multinucleated and micronucleated cells. Our results provide proof of concept that inhibition of the Aurora B–IN-box interaction is a viable strategy for interfering with CPC function *in vivo*.

## 2. Introduction

The chromosomal passenger complex (CPC), which consists of Aurora B kinase and the three non-enzymatic subunits inner centromere protein (INCENP) [1], survivin [2,3] and borealin/Dasra-B [4,5] orchestrates key events during mitosis [6]. INCENP acts as a scaffold for the CPC and also activates Aurora B. The INCENP N-terminus interacts with CPC targeting subunits survivin and borealin in a three-helix bundle [7]. INCENP also interacts directly with chromatin and the mitotic spindle via interactions with heterochromatin protein 1 (HP1) [8] and microtubules [2].

The C-terminus of INCENP contains the IN-box domain, which binds and activates Aurora B [9–13]. Upon INCENP binding, Aurora B phosphorylates a conserved threonine–serine–serine (TSS) motif located near the C-terminus of the IN-box [12,13]. This promotes CPC clustering, leading to *trans* phosphorylation of threonine 232 on the Aurora B activation loop and full kinase activation [14].

Aurora B is a member of a conserved family of serine/threonine kinases that, in vertebrates, contains a further two paralogues: Aurora A and C. Aurora A has key roles in mitotic entry, centrosome maturation and spindle organization [15], whereas Aurora C is essential for gametogenesis and appears to function in early embryogenesis rather than in somatic cells [16–19]. Because of their role in promoting accurate cell division, the Aurora kinases have been actively explored as targets for chemical inhibition in the treatment of cancer [20,21].

Aurora inhibitors constructed to date have all been designed to interfere with the activity of the ATP-binding pocket. Several relatively specific inhibitors have been obtained, including hesperadin, ZM447439 and AZD1152 [22–24]. However, in common with ATP-analogue inhibitors of many other kinases [25], these small molecules also exhibit off-target activity against a number of other kinases. Furthermore, it is relatively straightforward to isolate Aurora B mutants resistant to the inhibitors [26,27], and the utility of these compounds in the clinic may therefore be limited.

In this study, we have explored an alternative strategy for the inhibition of the CPC. As the interaction between INCENP and Aurora B is essential for activation and localization of the kinase, we have investigated the use of peptides and cyclic peptides to disrupt this interaction. We used successful completion of cytokinesis as a read-out for CPC activity, as even a small decrease in Aurora B activity impairs this process. Mild CPC hypomorphs capable of rescuing chromosomal alignment and other defects associated with CPC loss of function are unable to support normal cytokinesis in animal cells [28]. We find that a 75 aa peptide comprising most of the IN-box strongly interferes with CPC function *in vivo*. Further dissection of the interaction using short cyclic peptides yielded very modest but reproducible inhibitors of CPC function.

## 3. Results

### 3.1. Expression and processing of SICLOPPS constructs in HeLa cells

**Figure 1.**
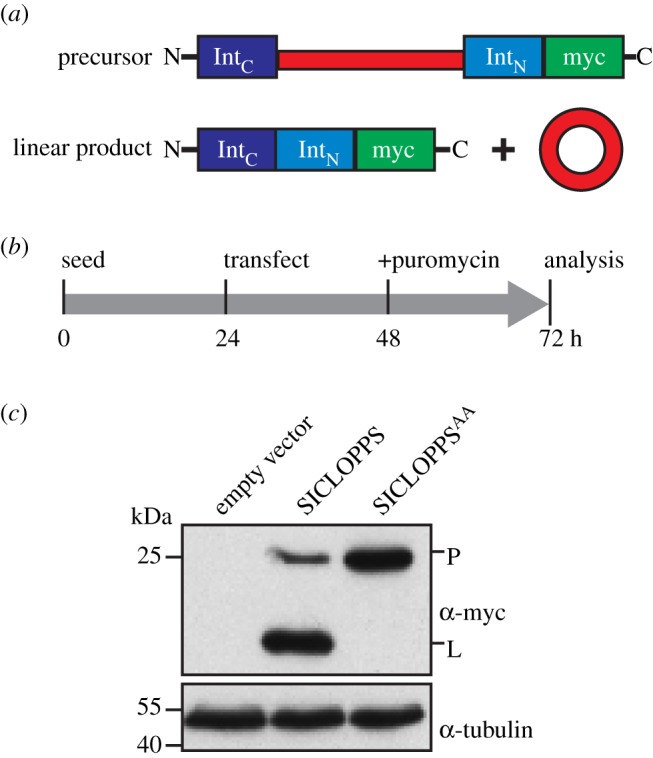
Expression and processing of SICLOPPS in HeLa cells. (*a*) Translation of SICLOPPS constructs produces a linear precursor that undergoes post-translational splicing to yield a linear product and a circularized peptide or protein (adapted from [30]). (*b*) Experimental timeline for testing SICLOPPS expression by transient transfection in this study. (*c*) Immunoblot detection of the SICLOPPS linear precursor (P) and the faster migrating linear product (L).

For the purpose of this study, we wished to genetically express the hINCENP IN-box domain and smaller fragments thereof as soluble peptides *in vivo*. As small, unmodified peptides tend to be unstable in cells, we sought to stabilize putative inhibitory peptides via head-to-tail circularization using split intein circular ligation of protein and peptides (SICLOPPS) [29]. This approach relies on the in-frame insertion of a nucleotide sequence encoding the desired protein or peptide as a linker between two halves of a split intein. The two halves of the split intein are oriented so that their post-translational splicing in *cis* leads to the excision of the linker region as a cyclic peptide (CP; figure 1*a*).

We opted to the use the naturally split DnaE intein from the cyanobacterium *Synechocystis* species PCC6803 (*Ssp*) [31] because it has been used in previous SICLOPPS library applications in prokaryotes and lower eukaryotes (e.g. [32,33]). As the *Ssp* DnaE intein had not previously been shown to circularlize peptides in human cells, we first assessed the expression and processing of a 3x*myc*-tagged *Ssp* DnaE SICLOPPS test construct.

For an initial test of the system, we transiently transfected HeLa cells with a construct expressing myc-tagged *Ssp* DnaE SICLOPPS with the linker peptide ‘CFGGSGGHPQFANA’, enriched for transfected cells by puromycin selection and examined whole cell lysates by immunoblotting to verify SICLOPPS construct expression and processing (figure 1*b*,*c*). At 48 h post-transfection, we could readily identify bands corresponding to the precursor and the faster migrating linear product. We note that throughout this study, we have not directly detected the cyclic peptides expressed in cells as the low abundance of these products often precludes their detection. However, based on a published study employing the *Ssp* DnaE intein for *in vivo* circular ligation in eukaryotes, detection of the linear product of intein splicing is a reliable reporter of cyclization [33].

In control experiments, cells were transfected with a construct expressing a double mutant version of the construct in which residues corresponding to T69 and H72 within the N-terminal *Ssp* DnaE intein were substituted with alanines (hereby referred to as SICLOPPS^AA^). These mutations have been previously shown to abolish splicing [34]. Indeed, only the precursor band was detected in lysates from cells expressing SICLOPPS^AA^ (figure 1*c*).

Together, these results demonstrate that the *Ssp* DnaE intein is readily expressed in HeLa cells and undergoes processing in a manner consistent with CP production.

### 3.2. Dominant-negative effect of the soluble INCENP IN-box

**Figure 2.**
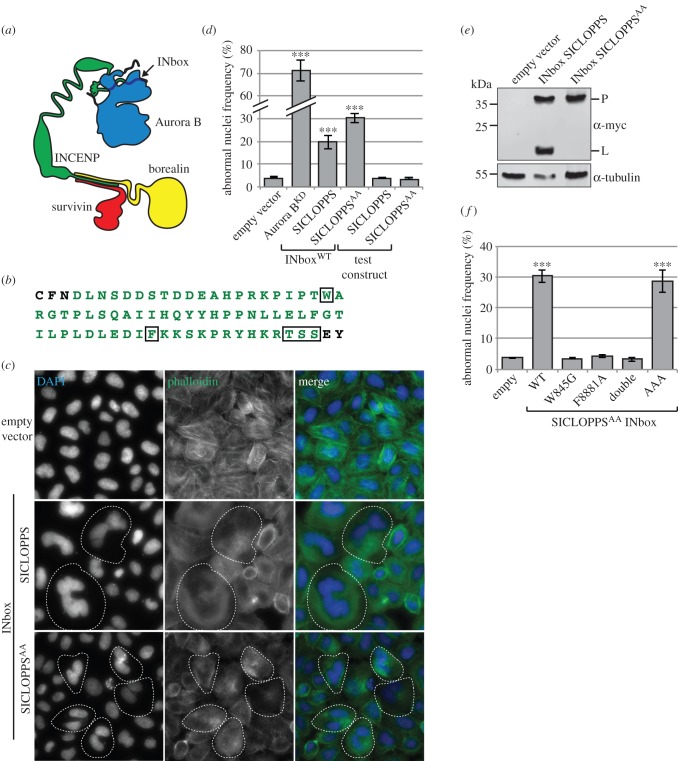
Soluble IN-box expression impairs CPC function. (*a*) Representation of the CPC highlighting the location of the IN-box (adapted from [6]). (*b*) Sequence of the soluble IN-box fragment inserted into the SICLOPPS construct containing IN-box residues (green) flanked by native *Ssp* DnaE extein residues (black). Boxed regions indicate location of mutations made to yield IN-box^W845G^, IN-box^F881A^, IN-box^dbl^ and IN-box^AAA^ constructs. (*c*) Representative micrographs of DAPI and rhodamine phalloidin-stained puromycin-selected cells transiently expressing soluble IN-box^WT^ constructs for 48 h. Dotted outlines indicate cells with nuclear morphological aberrations. (*d*) Quantification of abnormal nuclei frequency (ANF) in the same samples as the previous panel as well as for an Aurora B kinase-dead control. (*e*) Quantitative western blot detection of the SICLOPPS linear precursor (P) and the faster migrating linear product (L) in samples treated as in (*c*). Unspecific bands are marked with an asterisk. (*f*) Quantification of the ANF elicited by SICLOPPS^AA^ IN-box mutant constructs under identical conditions to those outlined in (*c*). (for (*c*) and (*f*): *n* = 3; more than 1000 cells per replicate; error bars: ±s.e.m.; *** indicates a significance of *p* < 0.001 between the sample and empty vector control as determined using the χ²-test).

In order to explore the consequences of inhibiting the Aurora B–IN-box interaction *in vivo* in HeLa cells, we expressed a soluble 75 amino acid hINCENP fragment spanning residues 825–894, which contains the IN-box. The location of the Aurora B-IN-box interface relative to the whole CPC is outlined in figure 2*a*. This IN-box fragment was inserted as a linker in the SICLOPPS construct and flanked with native *Ssp* DnaE extein residues in an attempt to enhance processing (figure 2*b*). As a control, we also expressed the Aurora B D218N kinase-dead mutant (hereby referred to as Aurora B^KD^), which has previously been shown to have a strong dominant-negative effect on CPC function [35]. Overexpression of Aurora B^KD^ had a very pronounced effect, eliciting an approximately 20-fold increase in the fraction of cells with abnormal nuclei (i.e. multinucleate and micronucleate cells; figure 2*c*,*d*). This increase in the abnormal nuclei fraction (ANF) was highly significant (*p* < 0.001, *n* = 3, *χ*^2^-test).

Microscopy of puromycin-enriched HeLa cells transiently expressing SICLOPPS IN-box^WT^ for 48 h also revealed a substantial increase in ANF, namely fivefold that of an empty vector control (*p* < 0.001, *n* = 3, *χ*^2^-test; figure 2*c*,*d*). Quantitative immunoblotting analysis revealed that ∼34% of the SICLOPPS IN-box^WT^ translation product was processed to yield the mature ligated extein, which is expected to correlate with IN-box^WT^ CP production (figure 2*e*). This dominant-negative effect was not due to the presence of the *Ssp* DnaE intein fragments. Under the same conditions, the SICLOPPS test constructs—whose linker has no primary sequence homology to the IN-box—had no effect on CPC function irrespective of their processing competency (figure 2*d*).

As processing of the SICLOPPS IN-box^WT^ construct was only of limited efficiency, this raised the question of whether the inhibition of CPC activity was due to the cyclic peptide, the linear precursor form or both. To distinguish between these possibilities, we constructed a processing-deficient version of the SICLOPPS IN-box^WT^ construct. When SICLOPPS^AA^ IN-box^WT^ was expressed in HeLa cells, it had an even more pronounced effect than the processing proficient SICLOPPS IN-box^WT^ construct (figure 2*c*,*d*). Interestingly, the mutant construct was approximately 35% more active at inhibiting the CPC than the wild-type construct, approximately equivalent to the processing efficiency of the wild-type construct. This suggests that the linear intein-flanked processing precursor may be responsible for inhibiting the CPC, rather than the circularized IN-box.

These experiments suggest that disruption of INCENP binding to Aurora B can effectively inhibit CPC activity, even in the presence of the endogenous wild-type proteins.

### 3.3. hINCENP W845 and F881, but not the TSS motif, are required for IN-box inhibition of CPC function *in vivo*

Although the most likely explanation of the inhibitory effect of the SICLOPPS and SICLOPPS^AA^ IN-box^WT^ constructs is that the IN-box fragment binds to Aurora B and displaces endogenous INCENP, other possibilities cannot be excluded. We, therefore, examined the effect of mutations in the IN-box that have previously been shown to interfere with INCENP binding to and/or activation of Aurora B. These mutants were introduced into the SICLOPPS^AA^ processing-deficient construct, as this gave the most robust inhibition of CPC function in our ANF assay.

Previous work had shown that mutation of the highly conserved residue equivalent to hINCENP W845 abolished the ability of INCENP to bind to Aurora B, and that this mutant form of INCENP could not support cell viability in DT40 cells [28]. Structural analysis of a *Xenopus* IN-box fragment bound to Aurora B predicted that this tryptophan was a critical determinant for the interaction between the two proteins [36]. In support of this, expression of SICLOPPS^AA^ IN-box^W845G^ in HeLa cells had no deleterious effect on CPC function in our assay (figure 2*f*). This strongly supports the notion that soluble IN-box inhibition of the CPC involves disruption of the interaction between endogenous INCENP and Aurora B.

Structural analyses had identified a second INCENP residue, corresponding to hINCENP F881, as likely to be essential for activation of the kinase [36]. A previous study had shown that truncated hINCENP lacking the region surrounding F881 could efficiently pull down Aurora B, albeit inactive, from cell lysates when these were coexpressed in Sf-9 cells [13]. Subsequent work in chicken DT40 cells showed that indeed an INCENP mutant lacking the corresponding phenylalanine could pull down Aurora B but could not rescue viability in the absence of wild-type INCENP [28]. Interestingly, expression of SICLOPPS^AA^ IN-box^F881A^ in HeLa calls also had no deleterious effect on CPC function (figure 2*f*). This suggests that, in the context of this IN-box fragment, F881 is required for binding of INCENP to Aurora B with sufficient affinity to interfere with binding of the endogenous proteins. As expected, a double mutant containing both mutations, SICLOPPS IN-box^W845G^/IN-box^F881A^ (referred to as SICLOPPS^AA^ IN-box^dbl^) also had no inhibitory effect on CPC activity (figure 2*f*).

The C-terminal region of the IN-box contains a threonine–serine–serine (TSS) motif that is phosphorylated by Aurora B and is required for the full activation of the kinase [12,13,37]. SICLOPPS^AA^ IN-box^AAA^ constructs in which the TSS motif was substituted with a stretch of alanines elicited a strong increase in ANF indistinguishable from the IN-box^WT^ constructs (figure 2*f*). This result suggests that the TSS motif is dispensable for Aurora B binding *in vivo*, consistent with previous observations [13,37].

### 3.4. Soluble IN-box causes the mislocalization of the CPC in mitosis

As INCENP is involved in both the activation and localization of the CPC during mitosis, we predicted that if soluble IN-box constructs disrupt the association of INCENP with Aurora B, then the localization of the kinase should be perturbed *in vivo*. To test this hypothesis, we examined the effect of expressing SICLOPPS^AA^ IN-box^WT^ and IN-box^dbl^, or an empty vector control, on the localization of Aurora B in mitotic HeLa cells.

**Figure 3.**
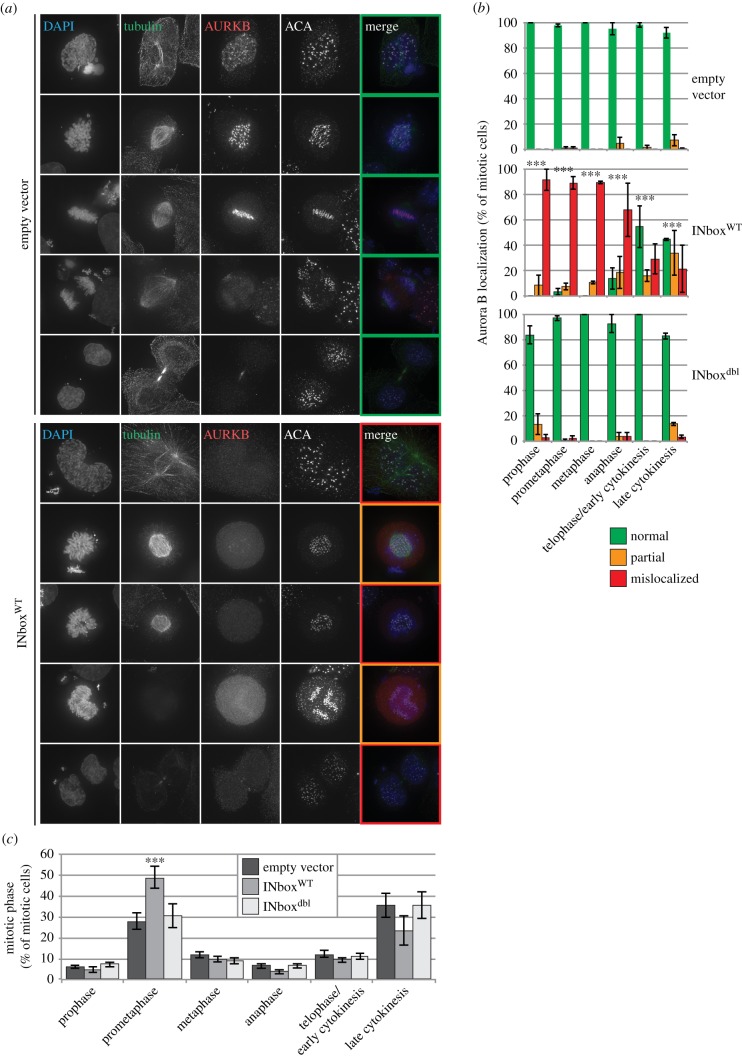
Soluble IN-box expression causes Aurora B mislocalization. (*a*) Representative micrographs showing the localization of Aurora B in puromycin-selected mitotic HeLa cells transiently transfected with either SICLOPPS^AA^ IN-box^WT^ or an empty vector control 48 h after transfection. DNA, the mitotic spindle and centromeres were detected using DAPI, anti-tubulin and ACA, respectively. (*b*) Quantification of Aurora B mislocalization during mitotic phases and cytokinesis in samples treated as in the previous two panels. (*n* = 3; more than 100 cells per replicate). Cells in which Aurora B was clearly visible in the cellular region matching canonical CPC localization [1] were scored as ‘normal’. Faint Aurora B accumulation in the correct region was scored as ‘partial’, as was clear signal only present in part of the region where it should be expected. All other cells were scored as ‘mislocalized’. Coloured boxes have been added around the merged images in panel A to show how these representative cells would be scored. (*c*) Distribution of cells in different stages of mitosis and cytokinesis in puromycin-selected HeLa cells transiently expressing SICLOPPS^AA^ IN-box constructs for 48 h. (*n* = 3; more than 100 cells per replicate; error bars: ±s.e.m.; *** indicates a significance of *p* < 0.001 between the sample and empty vector control).

Consistent with the hypothesis, the localization of Aurora B in mitotic cells was abnormal in cells expressing SICLOPPS^AA^ IN-box^WT^, but not IN-box^dbl^, when examined by immunofluorescence at 48 h post-transfection (figure 3*a*). Compared with control cells transfected with an empty vector, the localization of Aurora B was significantly affected in all stages of mitosis in cells transfected with IN-box^WT^ but not IN-box^dbl^ (*p* < 0.001 and *p* > 0.05, respectively, *n* = 3, Fisher's exact test; figure 3*b*). In the presence of IN-box^WT^, Aurora B was either completely or partially mislocalized in the vast majority of cells fixed in prophase to anaphase (figure 3*b*). The localization of Aurora B in cells in telophase or undergoing cytokinesis was less severely affected by IN-box^WT^ expression and normal localization could be observed in roughly half the cells scored. The appreciable drop in the severity of mislocalization in these later phases may be due to the use of transiently transfected constructs. That is, cells with lower soluble IN-box levels, which would occur owing to expression heterogeneity, may be less severely affected in both CPC localization and mitotic progression.

Interestingly, although cell samples each transfected with one of the three constructs had comparable mitotic indexes (empty vector: 4.3% ±0.6, IN-box^WT^ 4.3% ±0.7, IN-box^dbl^ 4.0% ±1.5) cells expressing the SICLOPPS^AA^ IN-box^WT^ construct exhibited a significant increase in prometaphase cells relative to the empty vector control (*p* < 0.001, *n* = 3, *χ*^2^-test; figure 3*c*). This increase in the number of cells in prometaphase suggests a chromosome alignment, but not mitotic checkpoint, defect.

In summary, the data presented thus far support a model where disruption of the interaction between INCENP and Aurora B is able to significantly interfere with CPC function *in vivo*.

### 3.5. INCENP IN-box cyclic peptide library screen

The Aurora B–IN-box interaction occupies an extended interface analogous to a crown on the small lobe of the kinase [36,38]. Although we are able to disrupt this interaction using a 75 amino acid residue construct, this is far from the short peptide interaction motifs that are typical starting points for the development of peptidomimetic small molecules. We therefore wished to explore whether smaller IN-box fragments could also dissociate the Aurora B–IN-box interaction.

To do this, we generated and assayed a library of circularized IN-box fragments ranging from four to eight residues. Each cyclic peptide also contained an invariable cysteine residue, derived from position +1 of the native extein, which is required for *Ssp* DnaE intein processing [39]. Thus, the library consisted of cyclic peptides ranging from pentamers to nonamers.

**Figure 4.**
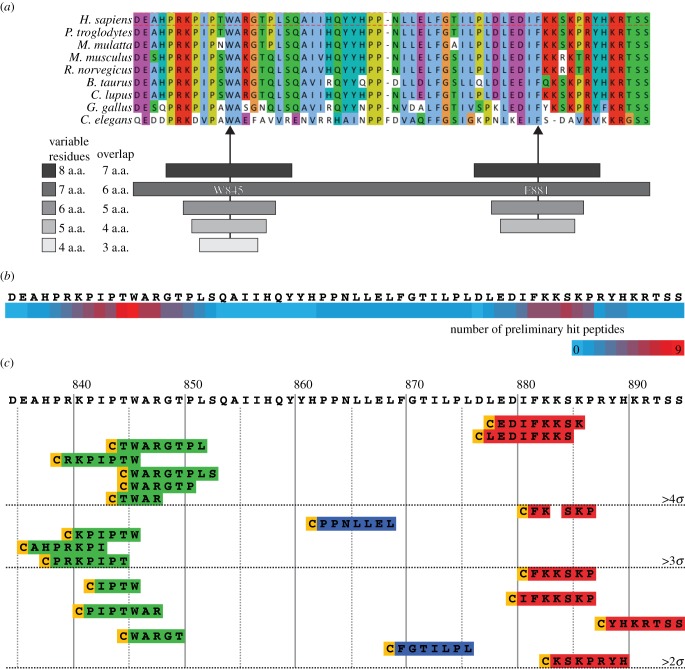
SICLOPPS library design and initial screen. (*a*) Upper half: conservation of the INCENP IN-box region used to design the library across a range of model organisms. Arrows highlight the residues equivalent to hINCENP W845 and F881. Lower half: coverage of the IN-box core and sub-libraries generated for this study. Each SICLOPPS construct was designed so that the circularized region consisted of an invariant cysteine residue followed by *n* variable residues based directly on the INCENP sequence. (*b*) Heat-map indicating the number of preliminary hits found that encompass a given IN-box residue. (*c*) Ranked and aligned sequences of the preliminary hits identified in the initial library screen comparing the ANF in puromycin-selected samples transiently expressing library constructs for 48 h to that of cells transfected with an empty vector. hINCENP residue numbers are indicated above the sequence of the IN-box fragment; the aligned constructs beneath the sequence are ranked in descending order. Horizontal dotted lines indicate standard deviation thresholds above the ANF found in samples transfected with an empty vector control.

To focus on IN-box residues most closely associated with the Aurora B N-terminal lobe, the library was designed to encompass INCENP residues 834–894—the region of the IN-box homologous to that used by Sessa *et al*., in their structural study of the Aurora B–IN-box complex [36] (figure 4*a*). The core library spanning this region consisted of octamers (seven variable residues plus the invariant cysteine). As we had shown that W845 and F881 are essential for IN-box binding to Aurora B, sub-libraries containing a range of cyclic peptide sizes were generated centered on those residues. Our intent in generating a range of cyclic peptides with similar sequences but different sizes was to vary the degree of conformational constraint on a given epitope. The core library and sub-libraries were designed so that adjacent constructs were offset by a single amino acid residue. The full library consisted of 97 members, 94 of which were successfully cloned in the SICLOPPS expression vector. The library peptide sequences can be found in electronic supplementary material, table S1.

The IN-box library was screened using the same ANF microscopy assay as the soluble IN-box constructs. A two-step strategy was used: a rapid first pass screen was used to screen out constructs with little or no effect, then the remaining candidate peptides were assayed in greater depth. In the first pass, the full library was screened twice independently. To normalize against any slight variation in ANF that might occur over successive cell passages, in each case, we compared the ANF increase of a construct relative to an empty vector control assayed in parallel, rather than raw ANF values. Constructs eliciting an average ANF increase greater than 2 standard deviations from that of the full set of empty vector control samples were carried forward into the next step. This cut-off criterion was met by 20 of the 94 library members (figure 4*b*). No constructs caused an average decrease in ANF greater than 2 standard deviations from the same control.

**Figure 5.**
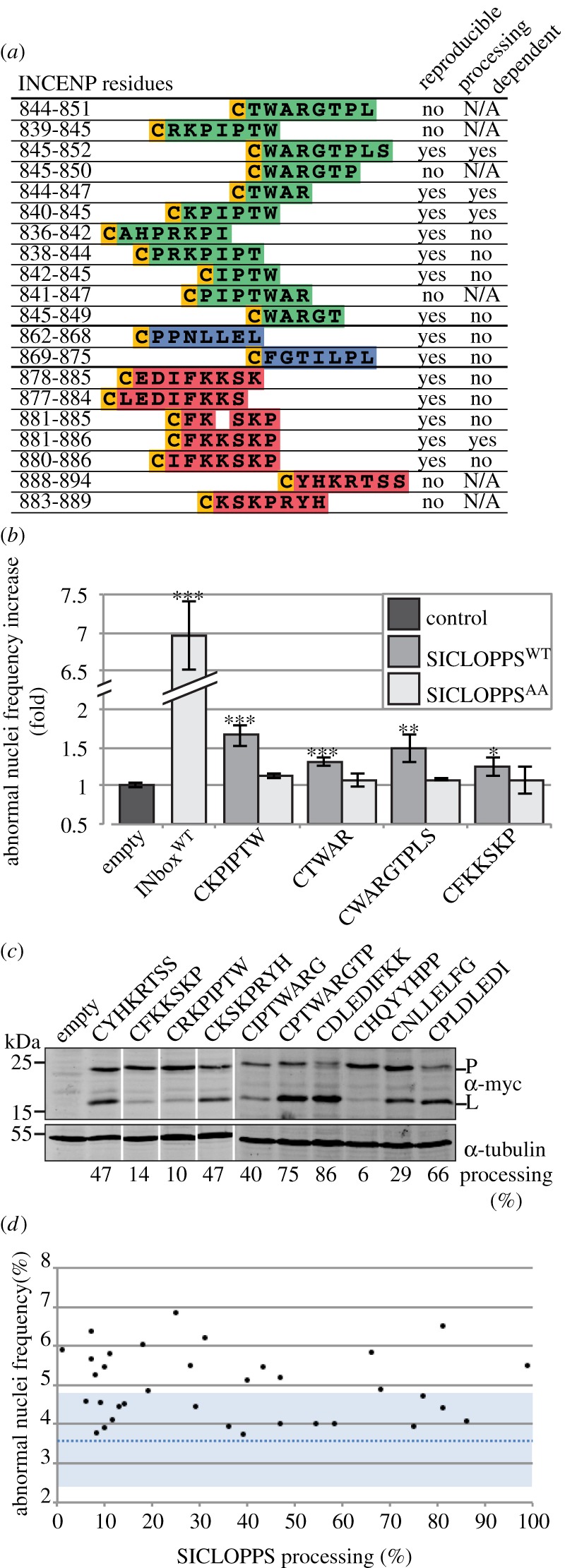
(*a*) Reproducibility and processing dependency of the hits identified in the initial screen. Reproducibility indicates that, when assayed in triplicate, the ANF increase elicited by a given processing-competent SICLOPPS construct was significantly different from that of an empty vector control (*p* < 0.05, *n* = 3, Mann–Whitney *U* test). Processing dependency means that the SICLOPPS^AA^ did not have a significant effect on ANF increase relative to the same control (*p* > 0.05, *n* = 3, Mann–Whitney *U* test). (*b*) ANF increase of the SICLOPPS^WT^ and SICLOPPS^AA^ versions of reproducible and processing dependent library constructs (*n* ≥ 3; error bars: ±s.e.m.; *, ** and *** indicate a significances of *p* < 0.05, <0.01 and <0.001, respectively, between the sample and empty vector control using Mann–Whitney *U* test). (*c*) Quantitative western blot detection of SICLOPPS processing in selected processing-competent library constructs. The extent of processing, indicated beneath each lane, is a measure of linear product (L) band intensity divided by the sum of the product and the precursor (P) bands. (*d*) Correlation between the extent of processing and the ANF elicited of selected IN-box library constructs, as measured by quantitative western blotting and fluorescence microscopy, respectively.

The initial hit constructs were then assayed in triplicate to verify whether their effects on ANF were reproducible, and whether their dominant-negative effect relied on peptide cyclization. Upon rescreening, 14 of the 20 initial hit constructs were found to produce reproducible effects (figure 5*a*). To test the cyclization dependency, putative hit constructs were assayed in parallel with matched processing-deficient SICLOPPS^AA^ mutants. Validated constructs whose matched SICLOPPS^AA^ version did not elicit a significant ANF increase relative to the empty vector control were deemed to be cyclization dependent. In these experiments, 4 of the 14 confirmed hit constructs were shown to require cyclization to produce their effect on ANF (figure 5*a*). Thus, the other 10 constructs appear to exert their effects as peptide aptamers, analogous to the SICLOPPS^AA^ IN-box construct.

Perhaps disappointingly, the strength of the inhibition observed for all constructs was modest. Although all peptides classified as ‘hits’ exhibited an ANF increase reproducibly over 2 standard deviations above the mean seen with the empty vector control, the maximum effect observed was an elevation of only 1.7× (figure 5*b*). For comparison, the SICLOPPS^AA^ IN-box^WT^ construct, caused an ANF of approximately 30% (figure 2*d*). This 7× ANF increase corresponds to greater than 38 standard deviations above the mean seen with the empty vector control (figure 5*b*).

We also wished to determine whether the magnitude of the observed effects on ANF depended on the efficiency of intein processing. In addition to the absolute requirement for a nucleophilic residue in extein position +1 [39], the efficiency of *Ssp* DnaE intein processing can also be modulated by other extein residues [40]. By definition, as we scanned across the IN-box sequence within the library, the residues present within the linker vary widely. To test whether there was a link between peptide activity and processing, a random sample of hit and non-hit peptides was assayed for processing efficiency and ANF increase in parallel (figure 5*c*,*d*). Processing efficiency was found to range between 1 and 99% (mean = 36.1%, s.d. = 28.36). No correlation was detected between SICLOPPS processing rates and peptide activity (Pearson's *r* = −0.11; *p* = 0.54; *n* = 35). This lack of correlation indicates that processing alone does not determine a construct's activity, suggesting that activity is dependent on the construct sequence. This observed heterogeneity of processing renders it extremely challenging to conduct quantitative comparisons of the activities of different hit peptides, or of to compare the activities of hit peptides against scrambled versions thereof using SICLOPPS methodology.

## 4. Discussion

These experiments show that disruption of the interaction between INCENP and Aurora B is a viable strategy for interfering with CPC function in cells. Of the two strategies explored here—using linear protein fragments and using cyclic peptides—the former option appears to be more effective at inhibiting the CPC. This is probably not solely due to the approximately 10× longer soluble IN-box peptide used compared with the constructs in our SICLOPPS library screen, as the circularized version of the longer (75 aa) IN-box peptide also appeared to be less efficient at inhibition the CPC than its linear counterpart.

Examination of the IN-box–Aurora B crystal structure suggests an obvious explanation for this effect [36,38]. In that structure, the IN-box encircles the small lobe of the kinase like a crown. If interactions around the perimeter of the small lobe can contribute to stabilizing the INCENP–Aurora B interaction, then it may well be that a linear peptide that can wind around the surface of the small lobe will be more efficient than a circular molecule which, in the case of our 75aa IN-box fragment would have to thread itself over the lobe to bind correctly and displace endogenous INCENP.

The failure of our cyclic peptide screen to identify strong inhibitors of the INCENP–Aurora B interaction may also be explained by the extended interaction surface seen in the crystal structure. For a small cyclic peptide to block INCENP binding, the peptide would have to occupy a localized high affinity site that is not readily displaced by the more extended endogenous polypeptide. Given that the single amino acid changes W845G and F881A can block INCENP binding to Aurora B, it seemed in principle possible that short high affinity peptides might displace the endogenous protein, but we did not find such peptides in our library. We note that, in contrast to the soluble IN-box, we found a small number of constructs that were more active in their processing-competent (i.e. SICLOPPS^WT^) forms. The IN-box–Aurora B interaction may be more accessible to smaller conformationally constrained probes. It is also possible that the bound intein moieties in the unprocessed constructs could in some cases interfere with the binding of shorter peptides such as those present in the library. Nevertheless, our cyclic peptide screen helps to establish parameters for future similar screens against Aurora B and other targets. We suggest, however, that in such screens, it may be more fruitful to target protein–protein interactions with less topologically constrained probes, provided that they are sufficiently stable *in vivo*.

The approach used here—probing the INCENP–Aurora B interaction using genetically encoded circular peptides—has several advantages over the alternative of chemically synthesizing cyclic peptides that can only be added exogenously to cells. Firstly, the chemical synthesis of such circular peptides is not routine, and is costly, particularly for the large numbers of cyclic peptides needed for library-scale screens. Secondly, once synthesized, such peptides are likely to have extremely variable solubility properties, given local variations in protein sequence. Thus, libraries of peptides are likely to require a variety of solubilization conditions, some of which may be toxic to cells. Thirdly, even when they are solubilized, it may be difficult to find a reproducible method for introducing the peptides into target cells.

On the other hand, our analysis has revealed a number of difficulties with the genetic encoding of cyclic peptides by the SICLOPPS strategy, in addition to the topological problems with circular peptides discussed above. Of these, perhaps the most significant is that of intein processing heterogeneity. Assessment of processing efficiencies within the library revealed extensive variability. Thus, despite the convenience of SICLOPPS for generating and delivering CP libraries, this processing heterogeneity of *Ssp* DnaE derived constructs limited the further assessment of our validated hits. For example, it is not clear how to conduct side-by-side comparisons of the effectiveness of pairs of cyclic peptides (e.g. wild-type and scrambled/mutant version) that are expressed and/or processed to different extents. This, coupled with the relatively low potency, led us to forego testing whether the activity of cyclization-dependent validated hit peptides containing residues W845 and F881 depended on those residues, as it did in the case of the full length IN-box construct. Furthermore, the average processing efficiency of the constructs assayed tended to be low. The effective size of our IN-box library in terms of effectively produced CPs may therefore have been smaller than anticipated. That a portion of constructs within a combinatorial SICLOPPS library will not be processed efficiently should also be taken into account when estimating the effective size and complexity of such libraries.

The dominant-negative effect of the soluble IN-box on CPC function is likely to be achieved by its interfering with the INCENP–Aurora B interaction. Our results therefore suggest that the INCENP–Aurora B interaction is a suitable target for an inhibitor approach. As demonstrated in earlier chicken DT40 INCENP knockout/replacement experiments, even a mild drop in CPC function is incompatible with cell proliferation and survival [37]. Thus, disrupting the Aurora B–IN-box interaction appears to be incompatible with cell survival.

The dominant-negative effect of the IN-box domain was similar but less pronounced than that of the Aurora B kinase-dead mutant. In both instances, endogenous Aurora B, which possesses basal activity, should be present in the cells. However, kinase-dead Aurora B might not only compete with endogenous Aurora B for binding to INCENP, but might also act as a competitive inhibitor by making non-productive binding interactions with potential substrates.

By mutagenizing the soluble IN-box construct, we were able to begin to map the structural requirements for its dominant-negative effect on CPC function. These requirements are likely to be similar as those for IN-box binding to Aurora B *in vivo*. Our findings that hINCENP W845 is necessary for the dominant-negative effect, and conversely that the TSS motif is dispensable, are in agreement with previously published observations on the IN-box–Aurora B interaction. Surprisingly, hINCENP F881 was also required for the INCENP fragment to interfere with CPC function in HeLa cells. Mutation of the equivalent INCENP residue to alanine in chicken DT40 cells still permits INCENP binding to Aurora B [28] and a construct lacking this residue can also pull down Aurora B *in vitro* [13]. Thus, the functional assay employed here appears to have greater sensitivity than conventional binding studies.

In summary, our results indicate that blocking the Aurora B–IN-box interaction could be a viable alternative to ATP-competitive inhibitors for Aurora B inhibition. The surfaces of Aurora B that interact with both hINCENP residues W845 and F881 may represent the most promising target sites for future drug design.

## 5. Material and methods

### 5.1. Tissue culture

HeLa Kyoto cells were maintained in Dulbecco's modified Eagle medium (Life Technologies) supplemented with 1% fetal bovine serum (SIGMA) and 1× penicillin/streptomycin (Life Technologies) at 37°C (5.0% CO_2_), and passaged as required.

### 5.2. Plasmids and transient transfection

All plasmids used in this study were generated in the pIRESpuro2 vector (Clontech). A synthetic C-terminally *3xmyc*-tagged *Ssp* sp. PCC6803 DnaE-based SICLOPPS ORF containing the insert CFGGSGGHPQFANA was custom synthesized by DNA2.0 (Menlo Park, CA, USA).

### 5.3. Antibodies

The following primary antibodies and dilutions were used for immunofluorescence: 1 : 500 rabbit polyclonal anti-Aurora B ab2254 (Abcam); 1 : 200 sheep polyclonal anti-alpha/beta tubulin ATNO2 (Cytoskeleton, Inc.); 1 : 200 human anti-centromere antibody (ACA; [41]). For western blotting, 1 : 15 000 mouse monoclonal B512 anti-αTubulin (SIGMA) and 1 : 1000 mouse monoclonal anti-Myc 9E10 (Covance) were used.

### 5.4. SICLOPPS IN-box library

The INCENP IN-box library was created in a modified version of pIRES puro2 vector from which the multiple cloning site (MCS) *AflII* site was removed by site-directed mutagenesis (oligos: 5′-CCGGTTAACAGGCCTATAGCGCTAGCTAGGCCGC-3′ and 5′-GCGGCCTAGCTAGCGCTATAGGCCTGTTAACCGG-3′). HPLC-purified oligos encoding each of the library members were purchased from Sigma-Aldrich (St Louis, MO, USA) and used to generate individual library inserts using the polymerase chain reaction (PCR) protocol described previously, but with the omission of the zipper step [30]. Inserts were amplified using the Expand High Fidelity PCR System (Roche) from a *NcoI*-digested pIRES puro2 SICLOPPS^WT^ template using a universal forward primer (5′-CCGAATTCGGATCCATGGTTAAAGTAATCGGC-3′) annealing to the MCS and a reverse primer (5′-GCCCGTATTCAACTGTAAGTATTTCAGTGCCAAAACTTAAGCA[variable]GCAATTATGGGCAATAGCC-3′) encoding the library insert, which spans the junction between the two intein halves and a *AflII* site present within the distal intein half. Library member amino acid sequences and primer nucleotide sequences are listed in electronic supplementary material, table S1. Library inserts were cloned into pIRES puro2 SICLOPPS^WT^ using *BamHI* and *AflII*. For transfection, library constructs were prepared using the transfection-grade Plasmid Mini Kit (QIAGEN) following the manufacturer's instructions. Protein-splicing deficient SICLOPPS^AA^ mutants of selected library constructs were generated by digestion with *BamHI* and *AflII* and insertion of the resulting fragment into pIRES puro2 SICLOPPS^AA^, which also lacks the MCS *AflII* site.

### 5.5. CPC function and SICLOPPS processing assays

HeLa Kyoto cells were seeded to a density of 5 × 10^4^ per well in 6-well plates containing uncoated 16 mm glass coverslips. Two days post-seeding, wells were transfected with 2 µg of plasmid using 6 µl X-tremeGENE 9 (Roche) following manufacturer's instructions. Twenty-four hours post-transfection, growth medium was replaced with fresh medium supplemented with 6 µg ml^−1^ puromycin, and samples collected for analysis 24 h later (i.e. 48 h post-transfection).

### 5.6. CPC functional assay

Coverslips were harvested, paraformaldehyde fixed, stained with rhodamine phalloidin (Life technologies) and mounted using Vectashield with DAPI (Vector Labs). Coverslips were scored visually on an Axioplan2 microscope using a Plan NEOFLUAR 40×/1,3 Oil objective (both Zeiss). Whole fields of view were scored for the frequency of cells with normal and abnormal (i.e. multinucleated, micronucleated and cells with chromatin bridges) nuclei for greater than or equal to 1000 cells. During library screening, to control for any fluctuation in background ANF, the ANF change for a given sample was calculated as the raw ANF value divided by that of samples transfected with empty pIRES puro2 vector in parallel on the same day.

### 5.7. SICLOPPS processing assay

SICLOPPS construct expression and processing were monitored by western blot. Cells were recovered from experimentally treated wells and lysed in 1× SDS-PAGE sample buffer. Samples were resolved on a 10% SDS-PAGE gel and transferred onto Hybond electrochemiluminescence (ECL) nitrocellulose membrane (GE Healthcare) by wet transfer overnight at 4°C using a constant voltage of 30 V. SICLOPPS expression and processing products were detected by ECL or quantitative (Li-Cor) western blotting.

## Supplementary Material

Supplementary Table 1 - INbox SICLOPPS library constructs and primers
