# Supplementary material for: Targeting the INCENP IN-box–Aurora B interaction to inhibit CPC activity in vivo
Source: Open Biol. 2014 Nov 12;4(11):140163. doi: 10.1098/rsob.140163 (PMC4248066; doi:10.1098/rsob.140163)
Supplement: Supplementary Table 1 - INbox SICLOPPS library constructs and primers [file rsob140163supp1.docx]

**Supplementary Table 1** – INbox SICLOPPS library constructs and primers

Sequence of the INbox library peptides and the primers used to generate these. The primer variable region is that encoding the cyclic peptide variable residues (i.e. excluding the invariant cysteine) and corresponds to the italicized region within the universal reverse primer sequence. The forward primer lays within the pIRES puro2 multiple cloning site and the reverse primer spans the junction between the two halves of the *Ssp* DnaE split intein. In designing primers, a sequence of two adjacent lysine residues (hINCENP 883-884) was mistakenly changed to a single lysine within one of the sub-libraries; the affected constructs are marked with an asterisk.

| **Universal primer** | **Primer Sequence (5'-3')** | | |
| --- | --- | --- | --- |
| Forward | CCGAATTCGGATCCATGGTTAAAGTAATCGGC | | |
| Reverse | GCCCGTATTCAACTGTAAGTATTTCAGTGCCAAAACTTAAGCA*(nnn)_n_*GCAATTATGGGCAATAGCC | | |
|  |  |  | |
| **Core library (7 variable residues)** | | | |
| **INCENP residues** | **Sequence** | **Primer variable region sequence (5'-3')** | |
| 834-841 | CDEAHPRK | CTTCCTAGGGTGGGCCTCGTC | |
| 835-842 | CEAHPRKP | AGGCTTCCTAGGGTGGGCCTC | |
| 836-843 | CAHPRKPI | GATAGGCTTCCTAGGGTGGGC | |
| 837-844 | CHPRKPIP | AGGGATAGGCTTCCTAGGGTG | |
| 838-845 | CPRKPIPT | GGTAGGGATAGGCTTCCTAGG | |
| 839-846 | CRKPIPTW | CCAGGTAGGGATAGGCTTCCT | |
| 840-847 | CKPIPTWA | GGCCCAGGTAGGGATAGGCTT | |
| 841-848 | CPIPTWAR | CCTGGCCCAGGTAGGGATAGG | |
| 842-849 | CIPTWARG | GCCCCTGGCCCAGGTAGGGAT | |
| 843-850 | CPTWARGT | GGTGCCCCTGGCCCAGGTAGG | |
| 844-851 | CTWARGTP | AGGGGTGCCCCTGGCCCAGGT | |
| 845-852 | CWARGTPL | CAGAGGGGTGCCCCTGGCCCA | |
| 846-853 | CARGTPLS | GCTCAGAGGGGTGCCCCTGGC | |
| 847-854 | CRGTPLSQ | CTGGCTCAGAGGGGTGCCCCT | |
| 848-855 | CGTPLSQA | GGCCTGGCTCAGAGGGGTGCC | |
| 849-856 | CTPLSQAI | GATGGCCTGGCTCAGAGGGGT | |
| 850-857 | CPLSQAII | GATGATGGCCTGGCTCAGAGG | |
| 851-858 | CLSQAIIH | GTGGATGATGGCCTGGCTCAG | |
| 852-859 | CSQAIIHQ | CTGGTGGATGATGGCCTGGCT | |
| 853-860 | CQAIIHQY | GTACTGGTGGATGATGGCCTG | |
| 854-861 | CAIIHQYY | GTAGTACTGGTGGATGATGGC | |
| 855-862 | CIIHQYYH | GTGGTAGTACTGGTGGATGAT | |
| 856-863 | CIHQYYHP | AGGGTGGTAGTACTGGTGGAT | |
| 857-864 | CHQYYHPP | AGGAGGGTGGTAGTACTGGTG | |
| 858-865 | CQYYHPPN | GTTAGGAGGGTGGTAGTACTG | |
| 859-866 | CYYHPPNL | CAGGTTAGGAGGGTGGTAGTA | |
| 860-867 | CYHPPNLL | CAGCAGGTTAGGAGGGTGGTA | |
| 861-868 | CHPPNLLE | CTCCAGCAGGTTAGGAGGGTG | |
| 862-869 | CPPNLLEL | CAGCTCCAGCAGGTTAGGAGG | |
| 863-870 | CPNLLELF | GAACAGCTCCAGCAGGTTAGG | |
| 864-871 | CNLLELFG | GCCGAACAGCTCCAGCAGGTT | |
| 865-872 | CLLELFGT | GGTGCCGAACAGCTCCAGCAG | |
| 866-873 | CLELFGTI | GATGGTGCCGAACAGCTCCAG | |
| 867-874 | CELFGTIL | CAGGATGGTGCCGAACAGCTC | |
| 868-875 | CLFGTILP | AGGCAGGATGGTGCCGAACAG | |
| 869-876 | CFGTILPL | CAGAGGCAGGATGGTGCCGAA | |
| 870-877 | CGTILPLD | GTCCAGAGGCAGGATGGTGCC | |
| 871-878 | CTILPLDL | CAGGTCCAGAGGCAGGATGGT | |
| 872-879 | CILPLDLE | CTCCAGGTCCAGAGGCAGGAT | |
| 873-880 | CLPLDLED | GTCCTCCAGGTCCAGAGGCAG | |
| 874-881 | CPLDLEDI | GATGTCCTCCAGGTCCAGAGG | |
| 875-882 | CLDLEDIF | GAAGATGTCCTCCAGGTCCAG | |
| 876-883 | CDLEDIFK | CTTGAAGATGTCCTCCAGGTC | |
| 877-884 | CLEDIFKK | CTTCTTGAAGATGTCCTCCAG | |
| 878-885 | CEDIFKKS | GCTCTTCTTGAAGATGTCCTC | |
| 879-886 | CDIFKKSK | CTTGCTCTTCTTGAAGATGTC | |
| 880-887 | CIFKKSKP | AGGCTTGCTCTTCTTGAAGAT | |
| 881-888 | CFKKSKPR | CCTAGGCTTGCTCTTCTTGAA | |
| 882-889 | CKKSKPRY | GTACCTAGGCTTGCTCTTCTT | |
| 883-890 | CKSKPRYH | GTGGTACCTAGGCTTGCTCTT | |
| 884-891 | CSKPRYHK | CTTGTGGTACCTAGGCTTGCT | |
| 885-892 | CKPRYHKR | CCTCTTGTGGTACCTAGGCTT | |
| 886-893 | CPRYHKRT | GGTCCTCTTGTGGTACCTAGG | |
| 887-894 | CRYHKRTS | GCTGGTCCTCTTGTGGTACCT | |
| 888-895 | CYHKRTSS | GCTGCTGGTCCTCTTGTGGTA | |
|  |  |  | |
| **sub-library (4 variable residues)** | | | |
| **INCENP residues** | **Sequence** | **Primer variable region sequence (5'-3')** | |
| 842-846 | CIPTW | CCAGGTAGGGAT | |
| 843-847 | CPTWA | GGCCCAGGTAGG | |
| 844-848 | CTWAR | CCTGGCCCAGGT | |
| 845-849 | CWARG | GCCCCTGGCCC | |
|  |  |  | |
| **sub-library (5 variable residues)** | | | |
| **INCENP residues** | **Sequence** | **Primer variable region sequence (5'-3')** | |
| 841-846 | CPIPTW | CCAGGTAGGGATAGG | |
| 842-847 | CIPTWA | GGCCCAGGTAGGGAT | |
| 843-848 | CPTWAR | CCTGGCCCAGGTAGG | |
| 844-849 | CTWARG | GCCCCTGGCCCAGGT | |
| 845-850 | CWARGT | GGTGCCCCTGGCCCA | |
| 877-882 | CLEDIF | GAAGATGTCCTCCAG | |
| 878-883 | CEDIFK | CTTGAAGATGTCCTC | |
| *879-885_ | CDIFKS | GCTCTTGAAGATGTC | |
| *880-886_ | CIFKSK | CTTGCTCTTGAAGAT | |
| *881-887_ | CFKSKP | AGGCTTGCTCTTGAA | |
|  |  |  | |
| **sub-library (6 variable residues)** | | | |
| **INCENP residues** | **Sequence** | **Primer variable region sequence (5'-3')** | |
| 840-846 | CKPIPTW | CCAGGTAGGGATAGGCTT | |
| 841-847 | CPIPTWA | GGCCCAGGTAGGGATAGG | |
| 842-848 | CIPTWAR | CCTGGCCCAGGTAGGGAT | |
| 843-849 | CPTWARG | GCCCCTGGCCCAGGTAGG | |
| 844-850 | CTWARGT | GGTGCCCCTGGCCCAGGT | |
| 845-851 | CWARGTP | AGGGGTGCCCCTGGCCCA | |
| 876-882 | CDLEDIF | GAAGATGTCCTCCAGGTC | |
| 877-883 | CLEDIFK | CTTGAAGATGTCCTCCAG | |
| 878-884 | CEDIFKK | CTTCTTGAAGATGTCCTC | |
| 879-885 | CDIFKKS | GCTCTTCTTGAAGATGTC | |
| 880-886 | CIFKKSK | CTTGCTCTTCTTGAAGAT | |
| 881-887 | CFKKSKP | AGGCTTGCTCTTCTTGAA | |
|  |  |  | |
| **sub-library (8 variable residues)** | | | |
| **INCENP residues** | **Sequence** | **Primer variable region sequence (5'-3')** | |
| 838-846 | CPRKPIPTW | CCAGGTAGGGATAGGCTTCCTAGGG | |
| 839-847 | CRKPIPTWA | GGCCCAGGTAGGGATAGGCTTCCT | |
| 840-848 | CKPIPTWAR | CCTGGCCCAGGTAGGGATAGGCTT | |
| 841-849 | CPIPTWARG | GCCCCTGGCCCAGGTAGGGATAGG | |
| 842-850 | CIPTWARGT | GGTGCCCCTGGCCCAGGTAGGGAT | |
| 843-851 | CPTWARGTP | AGGGGTGCCCCTGGCCCAGGTAGG | |
| 844-852 | CTWARGTPL | CAGAGGGGTGCCCCTGGCCCAGGT | |
| 845-853 | CWARGTPLS | GCTCAGAGGGGTGCCCCTGGCCCA | |
| 874-882 | CPLDLEDIF | GAAGATGTCCTCCAGGTCCAGAGG | |
| 875-883 | CLDLEDIFK | CTTGAAGATGTCCTCCAGGTCCAG | |
| 876-884 | CDLEDIFKK | CTTCTTGAAGATGTCCTCCAGGTC | |
| 877-885 | CLEDIFKKS | GCTCTTCTTGAAGATGTCCTCCAG | |
| 878-886 | CEDIFKKSK | CTTGCTCTTCTTGAAGATGTCCTC | |
| 879-887 | CDIFKKSKP | AGGCTTGCTCTTCTTGAAGATGTC | |
| 880-888 | CIFKKSKPR | CCTAGGCTTGCTCTTCTTGAAGAT | |
| 881-889 | CFKKSKPRY | GTACCTAGGCTTGCTCTTCTTGAA | |
|  |  | |  |
|  | | | |
